# Aberrant cerebellar Purkinje cell activity as the cause of motor attacks in a mouse model of episodic ataxia type 2

**DOI:** 10.1242/dmm.034181

**Published:** 2018-09-21

**Authors:** Esra Tara, Ariel Vitenzon, Ellen Hess, Kamran Khodakhah

**Affiliations:** 1 Dominick P. Purpura Department of Neuroscience, Albert Einstein College of Medicine, Bronx, NY 10461, USA; 2 Department of Pharmacology, Emory University School of Medicine, Atlanta, GA 30322-3090, USA

**Keywords:** Purkinje cells, Cerebellum, Episodic ataxia type 2, Tottering, SK channels

## Abstract

Many cerebellar-induced neurological disorders, such as ataxias and cerebellar-induced dystonias, are associated with abnormal Purkinje cell activity. In tottering mice, a well-established mouse model of episodic ataxia type 2 (EA2), cerebellar Purkinje cells are required for the initiation of motor attacks*.* How Purkinje cells contribute to the initiation of attacks is not known, and to date there are no reports on the activity of Purkinje cells during motor attacks in the tottering mice. Here, we show that tottering Purkinje cells exhibit high-frequency burst firing during attacks, reminiscent of other mouse models of cerebellar-induced motor dysfunction. We recorded the activity of Purkinje cells in awake head-restrained tottering mice at baseline, or during caffeine-induced attacks. During motor attacks, firing of Purkinje cells transformed to high-frequency burst firing. Interestingly, the extent to which the activity of Purkinje cells was erratic was correlated with the severity of the motor dysfunction. In support of a causal role for erratic activity in generating motor dysfunction, we found that direct infusion of the small conductance calcium-activated potassium (SK) channel activator NS309 into the cerebellum of tottering mice in the midst of an attack normalized the firing of Purkinje cells and aborted attacks. Conversely, we found that inducing high-frequency burst firing of Purkinje cells in wild-type animals is sufficient to produce severe motor signs. We report that erratic activity of wild-type Purkinje cells results in ataxia and dystonic postures. Moreover, this aberrant activity is the cause of motor attacks in the tottering mice.

## Abstract (precis)

**Summary:** Here, we report that in the well-established mouse model of episodic ataxia type 2, tottering, the severe episodic motor signs are caused by highly erratic activity of Purkinje cells.

## INTRODUCTION

Mutations in the *CACNA1A* gene, encoding for the P/Q-type voltage-gated calcium channel (Cav2.1), are known to result in neurological disorders, such as episodic ataxia type 2 (EA2), familial hemiplegic migraine type 1 (FHM1) and progressive spinal cerebellar ataxia type 6 (SCA6) (Ophoff et al., 1996; Zhuchenko et al., 1997). Each disorder is associated with different mutations in the *CACNA1A* gene that have differential effects on Cav2.1 function. For example, mutations in the *CACNA1A* gene leading to either truncation of the protein or loss of channel function result in EA2 (Guida et al., 2001; Jen et al., 2007; Ophoff et al., 1996, 1998; Rajakulendran et al., 2010). In this disorder, patients undergo attacks of severe ataxia (loss of motor coordination) and dystonia (sustained contractions of agonist and antagonist muscles) that are triggered by physical or emotional stress, caffeine or alcohol (Jen et al., 2007; Spacey et al., 2005). FHM1, on the other hand, is a rare autosomal-dominant disorder caused by gain-of-function missense mutations in the *CACNA1A* gene, and is characterized by episodes of severe migraines, hemiplegia, hemiparesis and, in some cases, progressive cerebellar ataxia (Ophoff et al., 1996). Several mouse models of EA2 (tottering, leaner, rolling Nagoya and rocker) and FHM1 (R192Q, S218L) exist, which carry loss-of-function mutations in the *Cacna1a* gene, in the case of EA2, and gain-of-function mutations, in the case of FHM1, and all exhibit many of the human signs (Fletcher et al., 1996; Mori et al., 2000; van den Maagdenberg et al., 2004, 2010; Zwingman et al., 2001).

Although each disorder is characterized by its own set of symptoms, they also share some in common. For example, about half of EA2 patients suffer from migraines and some FHM1 patients display progressive ataxia (Jen et al., 2007). Another common feature shared by these allelic disorders is that they all seem to involve cerebellar Purkinje cells in some way or another. Given that Purkinje cells highly express Cav2.1 channels, this is perhaps not surprising (Kulik et al., 2004). Much is known about the consequence of the mutations on channel function; however, how the mutations affect Purkinje cell activity *in vivo* is not known. We took advantage of the well-established mouse model of EA2, tottering, to scrutinize the role of Purkinje cells in the expression of motor attacks. Similar to the patients, tottering mice also carry a loss of function mutation (P601L) in the *Cacna1a* gene, leading to a ∼40% reduction in calcium current through the Cav2.1 channel (Wakamori et al., 1998). Moreover, similar to EA2 patients, tottering mice have episodes of severe ataxia and dystonia when exposed to physical or emotional stress, caffeine or alcohol (Raike et al., 2013b; Shirley et al., 2008).

In tottering mice, cerebellar Purkinje cells are required for the expression of attacks (Campbell et al., 1999; Raike et al., 2013a), and exhibit low-frequency oscillations under anesthesia (Chen et al., 2009). However, there is currently no information as to how the activity of Purkinje cells is altered during attacks of motor dysfunction. Here, using *in vivo* single-unit recordings in awake mice, we show that the activity of Purkinje cells during episodes of motor attack becomes extremely irregular. This is reflected as an increase in the interspike interval (ISI) coefficient of variation (CV), and the predominant firing rate (PFR) of tottering Purkinje cells during attacks. We have previously shown that the fidelity by which Purkinje cells encode information in their intrinsic activity requires precision of pacemaking in Purkinje cells (Walter et al., 2006). The more severe signs of the tottering mice during the attacks could be the consequence of greater loss of the information needed for motor coordination as a consequence of their erratic burst firing. In agreement with this hypothesis, we found that partially blocking calcium channels in the cerebellum of wild-type (WT) mice induced abnormal motor signs and, concurrently, high-frequency burst firing in Purkinje cells; and, conversely, that reducing burst firing by acutely injecting a small conductance calcium-activated potassium (SK) channel activator into the cerebellum of tottering in the midst of an attack aborted the attack.

## RESULTS

### The activity of Purkinje cells transforms to burst firing during attacks of motor dysfunction in tottering mice

**Fig. 1.**
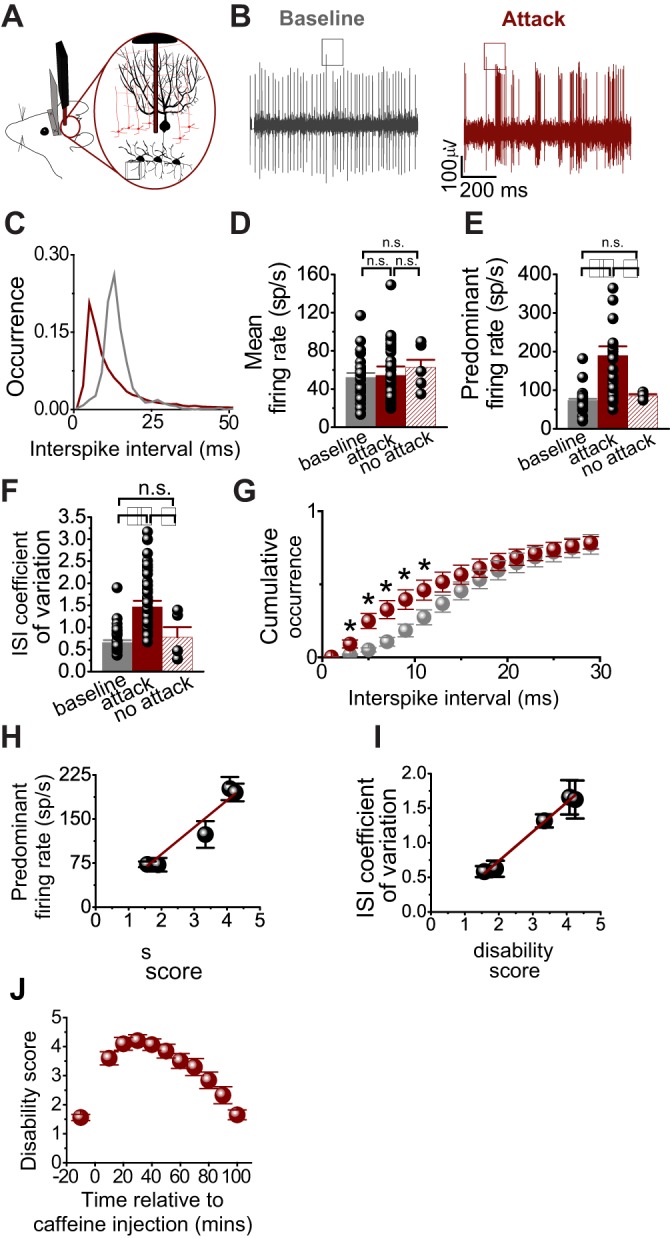
**Erratic firing of Purkinje cells correlates with the severity of motor dysfunction during caffeine-induced attacks.** (A,B) Activity of a typical Purkinje cell in the awake tottering mouse during baseline and caffeine-induced attacks. Asterisks indicate a complex spike, a hallmark of Purkinje cells. (C) ISI distribution histogram of a single Purkinje cell during baseline (gray, *n*=13) and attack (red, *n*=14). (D-F) Individual values and mean±s.e.m. of the firing rate (D), ISI CV (E) and PFR (F) of Purkinje cells at baseline, during caffeine-induced attacks or when no attacks occurred (*n*=5, N=2). (G) ISI cumulative distribution. *N*=6 mice. (H-I) Average ISI CV and predominant firing rate of Purkinje cells as a function of the average disability score. *n*=20, *N*=6. (J) Average disability score with respect to the time of caffeine injection. *N*=3. n.s., nonsignificant; *P<0.05, **P<0.001; one-way ANOVA with Tukey's multiple comparison test in D-F; two-way ANOVA with Sidak's multiple comparisons test in G.

We examined whether the motor attacks in tottering mice are accompanied by aberrant activity of Purkinje cells, by recording the activity of Purkinje cells in the awake head-restrained mice at baseline, and during caffeine-triggered episodes of motor dysfunction (Fig. 1A-C). We found that the average firing rate (number of action potentials in >5 min of recording divided by the total recording time) of Purkinje cells remained the same at baseline and during the attacks (baseline, 52±5 spikes/s, *n*=13; attack, 54±9 spikes/s, *n*=14, *N*=6, *P*>0.9; Fig. 1D). However, during the caffeine-induced attacks, the firing pattern of Purkinje cells transformed to high-frequency burst firing. This was evident in the significantly increased PFR (defined as the reciprocal of the mode of the ISI distribution histogram; baseline, 74±4 spikes/s; attack, 180±29 spikes/s; *P*<0.001 relative to baseline; Fig. 1E), and also in the ISI CV (calculated by dividing the standard deviation of the ISI by the mean ISI; baseline ISI CV, 0.66±0.06; attack, 1.47±0.14; *P*<0.001 relative to baseline; Fig. 1F), and an increase in the number of short ISIs in the ISI cumulative distribution which corresponded to high-frequency bursts (Fig. 1G). At the doses used, caffeine invariably produces an attack in the mice. In the rare instances when caffeine was injected but failed to induce attacks, the ISI CV and PFR were indistinguishable from baseline (PFR no attack, 85±5 spikes/s; *P*<0.05 relative to attack; ISI CV no attack, 0.78±0.23; *P*<0.05 relative to attack; Fig. 1D, *n*=5, *N*=2).

As mentioned earlier, the trigger-induced attacks in the tottering mice have been reported by many laboratories, including ours, to be highly stereotypic, and to progress in severity within the first 30 min and then slowly abate within 1 h or 2 h (Fig. 1J, *N*=3). We used this stereotypic response of the tottering mice to correlate the severity of the motor signs with the firing of Purkinje cells recorded at specific time points after the injection of caffeine. We found that as the severity of the attacks increased, so did the extent of burst firing in Purkinje cells (PFR versus disability, *R*^2^=0.95, *P*<0.003; ISI CV versus disability score, *R*^2^=0.99, *P*<0.0004; for electrophysiological recordings, *n*=20, *N*=6; for behavioral severity measurements, *N*=6; Fig. 1H,I).

### Restoring the regularity of Purkinje cell activity alleviates motor attacks

**Fig. 2.**
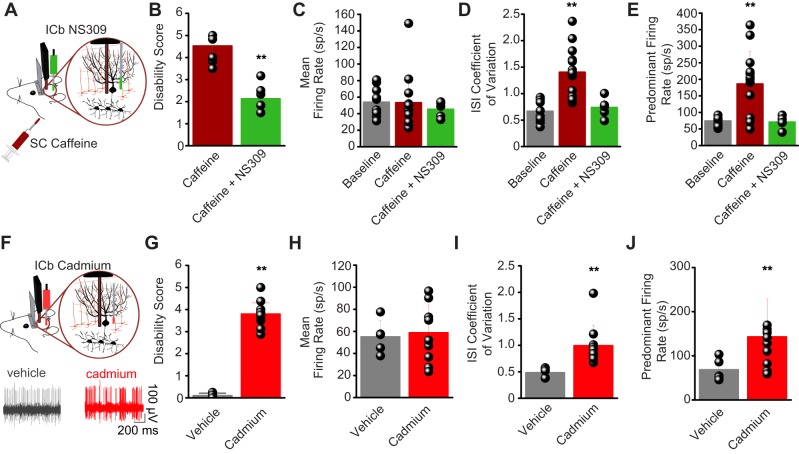
**Activation of SK channels aborts caffeine-induced attacks.** (A) Experimental design. ICb, intracerebellar; SC, subcutaneous. (B) Mean±s.e.m. of dyskinesia scores for caffeine-induced attacks (brown) and caffeine+NS309 infusion (green). *N*=8. **P<0.001, Student's *t*-test. (C-E) Individual values and mean±s.e.m. of the firing rate (C), ISI CV (D) and PFR (E) of tottering Purkinje cells at baseline (gray), during caffeine-induced attacks (brown, *n*=13, *N*=6) or with caffeine+NS309 infusion (green, *n*=5, *N*=3). **P<0.001, one-way ANOVA with Tukey's multiple comparison test. (F-J) Same as A-E, but for vehicle (gray, *N*=9) or cadmium (red, *N*=9). Inset shows representative raw traces of WT Purkinje cells after ICb vehicle (gray, *n*=5, *N*=2) or cadmium (red, *n*=10, *N*=3). **P<0.001, Student's *t*-test.

If erratic Purkinje cell activity is necessary for the expression of the severe motor signs in tottering, then restoring the regularity of Purkinje cell firing should abort the attacks and alleviate the episodic signs. To test this hypothesis, we directly injected 6,7-dichloro-1H-indole-2,3-dione 3-oxime (NS309), a potent and selective activator of SK channels (Strøbaek et al., 2004), into the cerebellum of the tottering mice in the midst of a caffeine-triggered attack. We targeted the SK channels because, in Purkinje cells, the calcium that enters the cell with each action potential activates SK channels (Womack et al., 2004; Womack and Khodakhah, 2002), which tightly regulates the duration of ISIs (Womack and Khodakhah, 2003). Indeed, in Purkinje cells, it has been shown that pharmacologically enhancing the activity of SK channels improves the precision of pacemaking and ataxia in the tottering mouse (Alvina and Khodakhah, 2010; Walter et al., 2006). At high concentrations, activators of SK channels can even decrease the firing rate of Purkinje cells (Womack and Khodakhah, 2003). Thus, an attack was triggered in tottering mice with caffeine; and, 10 min after caffeine injection, 0.5 μg NS309 was injected into the cerebellum with the aid of a pre-implanted cannula over a period of 15 min (Movie 1). Tottering mice have mild baseline ataxia, and the average baseline disability score of tottering mice under normal conditions was 1.57±0.1. During caffeine-induced attacks, the disability score increased to an average of 4.5±0.05 (Fig. 2B). Following injection of NS309, the disability score significantly reduced (2.1±0.3, *P*<0.001 compared with attack; *N*=8; Fig. 2B and Movie 1) to a value close to that under baseline conditions. Concurrently, cerebellar injection of NS309 restored the ISI CV and PFR of Purkinje cells to baseline values (PFR attack, 186±98 spikes/s, *n*=13, *N*=6; after NS309, 71±19 spikes/s, *n*=5, *N*=3; *P*<0.05; ISI CV attack, 1.4±0.48; after NS309, 0.74±0.19; *P*<0.05; Fig. 2D,E), without any change in the average firing rate (attack, 53±33 spikes/s; after NS309, 45±9 spikes/s; *P*>0.6; Fig. 2C). In the adult cerebellum, the Purkinje cells remain very sensitive to activation of SK channels, whereas the adult cerebellar nuclei neurons significantly reduce their reliance on these channels for their pacemaking (Alvina et al., 2016; Womack and Khodakhah, 2003), suggesting that in these experiments NS309 is likely to be predominantly affecting Purkinje cells rather than cerebellar nuclei neurons.

### Cadmium-induced erratic Purkinje cell activity is sufficient to induce motor dysfunction

If the observed aberrant cerebellar activity underlies the expression of episodic motor signs, inducing a similar pattern of Purkinje cell firing in WT mice should result in similar motor deficits. Chronic perfusion of the cerebella of WT mice with 8 μg/h CdCl_2_, which is known to induce Purkinje cell burst firing *in vitro* by altering its intrinsic pacemaking (Walter et al., 2006), produced severe motor dysfunction (disability scores: vehicle, 0.08±0.02, *N*=9; CdCl_2_, 3.8±0.21, *N*=9; *P*<0.001; Fig. 2G and Movie 2). Concurrently, the regular firing of Purkinje cells was transformed to burst firing similar to that seen in the tottering mice during attacks (PFR vehicle, 68±25 spikes/s, *n*=5, *N*=2; CdCl_2_, 142±85 spikes/s, *n*=10, *N*=3; *P*<0.05; ISI CV vehicle, 0.48±0.09; CdCl_2_, 0.99±0.38; *P*<0.001; mean firing rate vehicle, 55±15 spikes/s; CdCl_2_, 59±25 spikes/s; *P*>0.3; Fig. 2H-J). These findings support the prediction that pharmacologically inducing erratic Purkinje cell firing in WT mice can induce severe motor abnormalities comparable to those seen in the tottering motor attacks (Raike et al., 2013b). In these experiments, we cannot rule out that cadmium affected synaptic transmission throughout the cerebellum, or that it did not have an effect on any other neuron aside from the Purkinje cells. However, we have previously shown that pacemaking of cerebellar nuclei neurons in adult mice (Alvina et al., 2016), in contrast to that in juvenile mice (Alvina and Khodakhah, 2008), does not rely on calcium influx. Thus, in the experiments described above, cadmium will not have a major direct impact on the activity of the cerebellar nuclei neurons. Conversely, we have previously shown that both cadmium (Walter et al., 2006) and SK channel blockers (Womack and Khodakhah, 2003) directly affect the pacemaking of Purkinje cells and make them fire highly erratically. Thus, although we cannot rule out additional effects of cadmium on other neurons in the cerebellar cortex that might also affect the activity of Purkinje cells, there is no question that both agents make the activity of Purkinje cells highly irregular.

Similar results were obtained with acute injections of CdCl_2_ into the cerebella of a different set of WT animals where the mice showed ataxia and dystonia within minutes of injection of cadmium (vehicle disability score, 0.5±0.1, *n*=4, *N*=3; CdCl_2_, 4.7±0.1, *n*=4, *N*=3; *P*<0.001), indicating that the motor dysfunction associated with chronic cadmium perfusions were not due to the potential toxic effects of chronic exposure to cadmium, or the spread of cadmium to noncerebellar brain regions.

## DISCUSSION

The data presented here support the hypothesis that highly erratic activity of Purkinje cells during caffeine-induced attacks is the cause of motor abnormalities in tottering mice. This is in agreement with earlier studies that have shown that Purkinje cells are sufficient, and required, for the initiation of attacks in this mouse model (Campbell et al., 1999; Mark et al., 2011; Raike et al., 2013a), and extend them by revealing a tight correlation between the extent to which the activity of Purkinje cells is erratic with the severity of the motor abnormalities. Because a significant amount of sensory information converges on the cerebellum, one might consider the alternative hypothesis that the erratic activity of Purkinje cells in the tottering mice during the attacks simply reflects the abnormal sensory input caused by the signs. Three sets of observations make this unlikely and support the working hypothesis that the erratic activity of Purkinje cells causes the abnormal motor signs. The first is that on a number of occasions in the midst of an attack the high-frequency burst firing was noted when the animal was stationary and not moving at all. This argues against the possibility that it is the sensory feedback caused by overt ataxic movements that drives the Purkinje cells to burst firing. Second, inducing burst firing in the WT Purkinje cells was sufficient to cause comparable motor signs, and, conversely, terminating burst firing in the Purkinje cells aborted the motor attacks. Collectively, these observations favor the working hypothesis that erratic activity in Purkinje cells causes motor dysfunction, and that the severity of the motor dysfunction is correlated with the extent of erratic activity. Given this, and based on the data presented, pharmacologic restoration of Purkinje cell burst firing to normalcy could be a potential therapeutic approach for the management, and even aborting, of severe episodes of motor attacks in EA2.

The transformation of regular Purkinje cell activity to high-frequency burst firing during the episode of motor dysfunction described here in the tottering mouse has also been observed in other mouse models of cerebellar-induced motor dysfunction. For example, in the shRNA-based mouse models of early-onset dystonia (DYT1) and rapid-onset dystonia Parkinsonism (RDP), an aberrant output from the cerebellum is driven by high-frequency burst firing of Purkinje cells, and this abnormal activity is thought to be the cause of the dystonic phenotype in each model (Fremont et al., 2015, 2017). Aberrant Purkinje cell firing is also observed in the SCA6 mouse model, SCA6^84Q/84Q^ (Jayabal et al., 2016), as well as in *Itpr1*^−/−^ mice during dystonic episodes, and there too the pattern of firing of Purkinje cells is correlated with the severity of dystonia (Hisatsune et al., 2013). Although anesthesia is a major confounding factor, in the SCA2 mouse model SCA2-58Q, aberrant burst firing of Purkinje cells was seen in anesthetized mice (Egorova et al., 2016). Further corroborating our results, in a recent study, the authors could cause aberrant firing in WT Purkinje cells by application of SK channel blockers, and were able to normalize the firing of SCA2-58Q Purkinje cells using the KCa channel activator chlorzoxazone (Egorova et al., 2016), which we have previously proposed as a therapeutic agent for the treatment of ataxias (Alvina and Khodakhah, 2010). Thus, abnormal Purkinje cell activity seems to be often correlated with cerebellar-induced motor symptoms. In the tottering mice, dysfunction in the intrinsic activity of Purkinje cells is likely the cause of the abnormal firing observed *in vivo* (Walter et al., 2006)*.* Although dysfunction in synaptic transmission cannot be ruled out, the fact that in several mouse models of hereditary ataxias the selective expression of the mutant proteins only in Purkinje cells is sufficient to generate symptomatic mice suggests that abnormal activity of these cells could be the underlining cause of a number of cerebellar-induced motor disorders. It remains to be established, however, how mutations in each of these proteins disrupt Purkinje cell activity.

### Tottering as an EA2 mouse model

Although the tottering mice have been used as a mouse model of a number of different disorders, including paroxysmal nonkinesigenic dyskinesia, dystonia and absence epilepsy (Fletcher et al., 1996; Raike et al., 2013a; Shirley et al., 2008), they are perhaps best representative of EA2. The tottering mice were discovered in 1957 in The Jackson Laboratories based on their severe motor signs: wide gate, ataxia and absence seizures (Green and Sidman, 1962; Noebels and Sidman, 1979). Later, they were found to harbor a spontaneous missense mutation (P601L) in the *Cacna1a* gene (Green and Sidman, 1962) that results in the loss of function of the Cav2.1 channel. When unequivocally ascertained, in EA2 patients, the missense, truncating or CAG repeat mutations identified so far have been shown, or are predicted, to be loss-of-function mutations, and only in a small fraction of cases (∼20%) the nature of the mutations is not yet established (Guida et al., 2001; Jodice et al., 1997; Spacey et al., 2005). In all cases examined, mice that harbor loss-of-function mutations in the *Cacna1a* gene have been found to exhibit characteristic signs of EA2, rather than those of the other allelic disorders, FHM1 or SCA6 (Fletcher et al., 1996; Mori et al., 2000; Zwingman et al., 2001). Conversely, mice which harbor missense mutations in the *Cacna1a* gene that lead to a gain of function (as seen in FHM1 patients) exhibit many of the human FHM1 signs (van den Maagdenberg et al., 2004, 2010). SCA6, the third disorder associated with mutations in the *CACNA1A* gene, is characterized by cerebellar ataxia and progressive cerebellar degeneration, and is caused by CAG repeat expansions in the *CACNA1A* gene, resulting in an aberrant polyglutamine chain in the C-terminus of Cav2.1 channels (Watase et al., 2008; Zhuchenko et al., 1997). Mouse models carrying aberrant copies of the CAG repeat in the *Cacna1a* gene recapitulate the human SCA6 signs (Zhuchenko et al., 1997).

Thus, based on the discussions above, it is clear that the tottering mouse is a faithful and perhaps invaluable model of EA2. Given that, as reported here, pharmacologic restoration of Purkinje cell burst firing to normalcy aborts attacks and alleviates the caffeine-induced motor abnormalities in the tottering mice, it is plausible that a similar approach might constitute a potential therapeutic approach for the management of attacks in patients suffering from EA2.

## MATERIALS AND METHODS

### Animals

Experiments were performed on 3- to 9-month-old male and female tottering mice on a C57/Bl6 background, in accordance with the guidelines set by the Albert Einstein College of Medicine. Tottering (*tg*/*tg*) and heterozygous (*tg*/+) mice were a kind gift from Dr Ellen Hess (Emory University, Atlanta, GA, USA). The *tg*/+ mice were bred in-house, and *tg*/*tg* mice were born in a Mendelian fashion.

### Induction of attacks

Attacks were induced by subcutaneous injections of 10 mg/kg caffeine (Sigma-Aldrich, St. Louis, MO, USA), a commonly used paradigm to trigger attacks in tottering mice (Fureman et al., 2002). Mice were videotaped 10 min prior, and 40 min following, attack induction. For the experiments in Fig. 1J, 30 s videos were taken every 10 min starting 10 min prior to inducing an attack and ending 100 min after attack induction.

### *In vivo* electrophysiology

All surgical procedures were performed under isofluorane anesthesia (5% induction and 2% maintenance). A custom-made ‘L’-shaped bracket was fixed onto the skull with three bone screws (Plastics One, Wallingford, CT, USA) and dental cement (M&S Dental Supply, Jamaica, NY, USA). A recording chamber 3 mm in diameter was drilled in the skull above the cerebellum, surrounded with dental cement, and covered with surgi-foam (Ethicon, Somerville, NJ, USA) and bone wax (Ethicon). Following surgery, mice were allowed to recover for 1 week prior to the recording sessions.

To record neural activity, the mouse's head was immobilized by fixing the previously implanted head bracket to the stereotaxic apparatus with a screw, with the mouse standing on a flat surface, allowing the experimenter to observe its movements. The surgi-foam and bone wax were removed, and the recording chamber was filled with agar. To reduce the probability of stress-triggered attacks in the tottering mice when they were affixed to the stereotaxic apparatus, each mouse was conditioned to the apparatus for 30-60 min/day for 17-14 days, as needed. Single-unit activity of Purkinje cells was recorded using a platinum-quartz electrode (2-3 MΩ, Thomas Recording, Giessen, Germany), which was advanced into the cerebellum until the Purkinje cell layer was reached. For electrical reference, we either put a screw above the cortex, or placed a wire in the saline bathing the craniotomy. Purkinje cells were identified by the brief pause in their activity following each complex spike, their location and their characteristic firing rate *in vivo* (Hoebeek et al., 2008). Each cell was recorded for at least 5 min and attributed to the attack condition when motor signs could visually be identified and quantified. To aid in accurate quantification of the severity of motor abnormalities, mice were removed from the stereotaxic apparatus periodically and placed in an open cage and videotaped, and the motor signs were quantified as described below. If caffeine injection failed to induce an attack, cells recorded within a 50 min time window following caffeine administration were attributed to the no attack condition. Extreme care was taken to reduce stress, and the motor behavior of mutant mice was closely monitored to detect spontaneous/stress-triggered attacks, which, despite the conditioning described above, can occur infrequently under these experimental conditions. Cells were only classified as baseline when mice exhibited no motor abnormalities for at least 10 min before and after recording.

Neural signals were band-pass filtered (80 Hz–20 kHz), amplified (2000×) and digitized (20 kHz) using a data acquisition card (Purkinje cellI-MIO-16XE) and in-house written software based on LabView (National Instruments, Austin, TX, USA). Waveforms were sorted using Offline Sorter software (Plexon, Dallas, TX, USA), using principal component analysis.

### Cerebellar injections and chronic perfusion

A single injection cannula (Plastics One) was stereotaxically implanted at midline [distance from Bregma: anteroposterior (AP), −6.90 mm; dorsoventral (DV), 2 mm] for NS309 (Tocris, Bristol, UK) injections, and a double injection cannula [mediolateral (ML), ±0.75 mm; AP, −6.90 mm; DV, 2 mm] for injection of CdCl_2_. Following recovery, a total volume of 5 μl of the desired solution was injected over a period of 15 min using an automated pump (World Precision Instruments, Sarasota, FL, USA). The behavior of each mouse before and after drug perfusion and injection was documented by video recordings. Cerebellar chronic perfusions were performed as described previously (Calderon et al., 2011). Briefly, bilateral cannulas (Plastics One) were stereotaxically implanted (AP, 0.74 mm; ML, 1.5 mm; DV, 4 mm) and connected to two osmotic pumps (model 1007D, 0.25 μl/h, Alzet, Cupertino, CA, USA), which were placed under the skin on the back of the mice.

### Disability rating scale

The severity of motor dysfunction was quantified according to a previously published scale (Weisz et al., 2005): 0, normal; 1, slightly slowed or abnormal movements; 2, mild impairment, limited ambulation unless disturbed; 3, moderate impairment, limited ambulation when disturbed, frequent abnormal postures; 4, severe impairment, many abnormal postures; 5, almost no ambulation, sustained abnormal movements. Videos were scored by three to four observers who were blind to the experimental procedure and their scores were averaged.

### Statistical analysis

All statistical tests were analyzed using GraphPad Prism software v.7 for Windows. All data are reported as mean±s.e.m. When two conditions were compared, we used the unpaired Student's *t*-test. When three conditions were compared, we used one-way ANOVA with Tukey's multiple comparison test. In Fig. 1G, we used two-way ANOVA with Sidak’s multiple comparisons test. Only data with *P*<0.05 were considered as statistically significant. Note that throughout the text ‘*n*’ refers to the number of cells or trials, whereas ‘*N*’ refers to the number of mice.
